# First Presentation of Pheochromocytoma As Acute Myocarditis in Otherwise Young Healthy Man

**DOI:** 10.7759/cureus.48554

**Published:** 2023-11-09

**Authors:** Ano Shalomi, Ramanathan Ramesh

**Affiliations:** 1 Medicine, Teaching Hospital, Batticaloa, Batticaloa, LKA; 2 General Medicine, Teaching Hospital, Batticaloa, Batticaloa, LKA

**Keywords:** chromogranin, palpitation, myocarditis, hypertension, pheochromocytoma

## Abstract

Phaeochromocytoma is indeed a rare and frequently misunderstood neuroendocrine tumor originating from chromaffin cells in the adrenal medulla. Its clinical presentation often includes paroxysmal hypertension, palpitations, headache, and diaphoresis, which can easily be mistaken for common medical conditions. Timely diagnosis and precise localization are paramount for ensuring the best possible outcomes for patients.

In this case report, we describe an unusual presentation of phaeochromocytoma in a 36-year-old man who presented with acute myocarditis. This atypical manifestation underscores the diagnostic challenges associated with phaeochromocytoma, as its symptoms can mimic various other cardiac and non-cardiac conditions. Vigilant clinical evaluation and a multidisciplinary approach are essential for promptly recognizing and managing such cases, thus optimizing patient care and prognosis.

## Introduction

Myocarditis, an inflammatory condition of the heart muscle, can result from a variety of causes, including viral infections, autoimmune processes, and toxic exposures [1]. Emerging evidence suggests that prolonged exposure to high levels of catecholamines in patients with uncontrolled phaeochromocytoma can lead to direct myocardial injury and inflammation, triggering myocarditis [2]. Takotsubo cardiomyopathy, also known as “broken heart syndrome” or stress cardiomyopathy, is a condition where the heart muscle becomes temporarily weakened or stunned, typically in response to severe emotional or physical stress. It’s important to note that both Takotsubo cardiomyopathy and pheochromocytoma-induced myocarditis can lead to similar symptoms, such as chest pain and heart-related issues. However, the underlying mechanisms and causes differ.

The clinical presentation of phaeochromocytoma-induced myocarditis often includes symptoms such as chest pain, dyspnea, palpitations, and signs of congestive heart failure, which may be misattributed to hypertension or other cardiac conditions. The overlapping clinical manifestations and diagnostic challenges are discussed, emphasizing the importance of considering phaeochromocytoma in the differential diagnosis of unexplained myocarditis, particularly in young and otherwise healthy individuals [3].

## Case presentation

A 36-year-old man who was previously unevaluated, presented with burning type of central chest pain with severe vomiting for 12 hours duration. He appeared to be healthy until he was admitted to our hospital with the above symptoms. There was a history of fever three days before admission. His past medical and surgical history was not significant. There were not any significant diseases running in his family. He used alcohol occasionally; his last alcohol consumption was one month back.

On admission, his Glasgow coma scale (GCS) was 15/15. His oxygen saturation was 99% in room air, heart rate was 96 beats per minute, and his blood pressure was 100/60 mmHg. On his respiratory examination, breath sounds were equal on both sides, vesicular in nature and no added sounds were heard. Abdominal and neurological examinations were unremarkable. His ECG showed widespread PR depression, without any other abnormalities, and his high sensitivity troponin I was markedly high (25310, Ref-Male: < 12 ng/L). He was initially treated with subcutaneous enoxaparin, antiplatelets, and statin for acute coronary syndrome.

**Figure 1:**
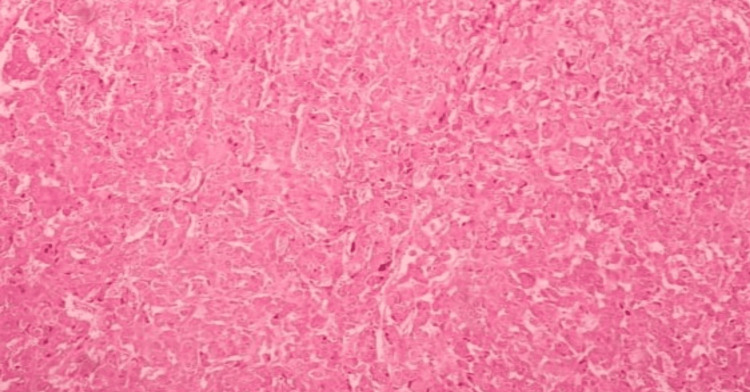
Histological appearance of pheochromocytoma (Hematoxylin and Eosin staining).

**Figure 2:**
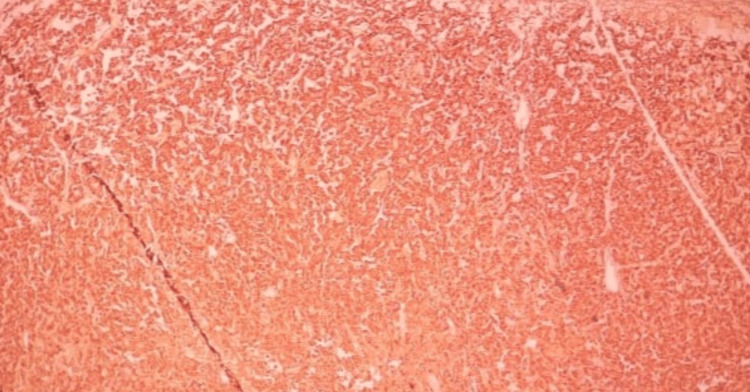
Histological appearance of pheochromocytoma (Chromogranin staining).

**Figure 3:**
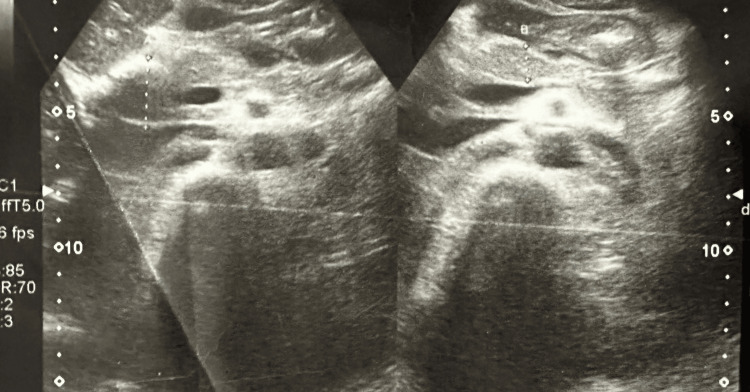
Ultrasound evidence of pheochromocytoma.

The next day morning, he developed difficulty in breathing, and his saturation dropped to 88% in room air. His pulse rate was 148 per minute and blood pressure 105/70 mmHg. His bedside 2D echocardiogram showed global hypokinesia and ejection fraction was 20%. Initially, this was suspected as an acute coronary syndrome or viral myocarditis, however, during the treatment process, he developed intermittent restlessness, fluctuating blood pressure, and heart rate with echocardiographic changes raising the possibility of pheochromocytoma. Diagnosis of pheochromocytoma confirmed by metanephrine levels and tumor confirmed by contrast-enhanced CT (CECT) scan of the abdomen and pelvis. His CECT scan showed a well-defined solid mass measuring 4.5 cm x 4.1 cm x 5.7 cm in size seen arising from the left suprarenal gland. We initiated treatment with the guidance of an endocrinologist and referred the patient for surgical excision, as it is the best treatment option [4]. He underwent open left pheochromocytoma excision and histology confirmed the diagnosis of pheochromocytoma (Figures 1-2). His routine investigation results are shown in Table 1, and specific laboratory investigations are in Table 2. Ultrasound evidence of pheochromocytoma is shown in Figure 3.

**Table 1:** Routine investigation results. WBC: white blood cells; HGB: hemoglobin; PLT: platelet; AST: aspartate aminotransferase; ALT: alanine aminotransferase; PT: prothrombin time; INR: international normalized ratio; CRP: C- reactive protein; ESR: erythrocyte sedimentation rate; UFR: urine full report; CPK: creatine kinase; FBS: fasting blood sugar; TSH: thyroid stimulating hormone; pre-op: preoperative; On D: on discharge.

Investigations	Day 1	Day 4	Day 7	Pre-op	On D	Reference range
WBC/mm^3^	20.75 x 10^3^	14.94 x 10^3^	15.97 x 10^3^	9.96 x 10^3^	10.4 x 10^3^	4-10 x 10^3^/µL
Neutrophil (%)	80.6	72.4	74.5	62.5	72.5	50-70%
Lymphocytes (%)	10.4	18.0	16.3	26.7	15.2	20-40%
Eosinophils (%)	0.1	0.1	1.1	3.4	2.6	0.5-5%
HGB (g/dL)	15.9	13.8	12.7	11.9	10.7	11-15
PLT (mm^3^)	345x 10^3^	210x 10^3^	286x 10^3^	326x 10^3^	178x 10^3^	150-450 x10^3^
Serum creatinine (μmol/ L)	117	126	158	66	93	62-115
Blood urea (mmol/ L)	9.4	14.2	10.0	4.6	3.6	1.8-6.3
Serum Na+ (mmol/ L)	139	145	142	138	133	136-145
Serum K+ (mmol/ L)	3.9	4.7	4.1	4.6	3.8	3.5-5.1
AST(U/L)	174	109	46	48	60	15-37
ALT(U/L)	64	93	103	114	91	12-78
Albumin (g/L)	34	26	36	34	33	34-50
PT/INR	1.37	-	1.37	0.94	1.08	-
CRP (mg/L)	17	43	12	2.5	5.6	0-5
Amylase (U/L)	85	-	-	-	-	25-115
ESR (mm/1^st^ hour)	10	-	18	-	-	-
UFR pus cells	-0-1	-	-	nil	-	-
UFR albumin	Nil	-	-	nil	-	-
UFR red cells	Nil	-	-	nil	-	-
CPK(U/L)	-	-	151	-	-	39-308
Calcium (mmol/l)	2.5	-	2.3	-	-	2.1-2.5
Phosphate(mmol/l)	-	-	1.2	-	-	0.8-1.6
FBS (mmol/L)	6.2	-	6.2	-	-	3.4-5.5
TSH (0.46-4.68) (mIU/L)	-	1.59	-	-	-	0.46-4.68
Free T4 (10.0-28.2) (pmol/ L)	-	27.6	-	-	-	10-28

**Table 2:** Specific laboratory investigations. VMA: vanillylmandelic acid

Test	Result	Reference range
24-hour urine metanephrine	>2000 mg/day	<350 mg/day
24-hour VMA	47.3 mg/24 h	<13.6 mg/24 h
Chromogranin A (CgA)	3129.7 mg/L	<100 mg/L

Our patient successfully underwent surgery, recovered postoperatively, and was discharged with appropriate follow-up care.

## Discussion

He was a 36-year-old patient who had not been previously evaluated, and presented with epigastric pain and vomiting. Upon further questioning, he revealed that he had experienced infrequent episodes of the same symptoms. Initially, our differential diagnoses included gastroesophageal reflux disease, acute pancreatitis, peptic ulcer disease, or cardiac disease.

However, due to his serum amylase levels being within the normal range, an ECG showing sinus tachycardia, a positive troponin I level, and global hypokinesia with an ejection fraction of 20% in the echocardiogram, we arrived at a working diagnosis of acute coronary syndrome or viral myocarditis. Even though an endomyocardial biopsy for a definitive diagnosis of myocarditis may not be feasible in resource-poor settings, the diagnosis of myocarditis was made based on clinical presentation and investigations, such as elevated cardiac markers and 2D echo findings. During the ward stay, we observed intermittent restlessness, which prompted consideration of meningoencephalitis or delirium. However, his investigations did not support these diagnoses.

The patient developed intermittent hypertensive episodes with improvements in echocardiographic findings, leading us to consider the rare possibility of pheochromocytoma. Imaging investigations were conducted to identify the lesion. His ultrasound scan revealed a left suprarenal mass measuring 4.1 cm x 4.7 cm in size, which was further confirmed by a CECT scan, showing a well-defined solid mass measuring 4.5 cm x 4.1 cm x 5.7 cm arising from the left suprarenal gland. Blood samples were taken for CgA levels, urine was collected for 24-hour urine metanephrine, and 24-hour vanillylmandelic acid was also tested. Based on the results of these investigations, the diagnosis of pheochromocytoma was established.

Myocarditis due to pheochromocytoma has been reported for decades, even though it is a rare presentation. Like previously reported cases, our patient also initially presented with features of cardiac disease [5]. With inward close monitoring and previous knowledge from case reports, our differential diagnosis shifted toward pheochromocytoma. According to studies, cardiac involvement in pheochromocytoma is frequent and may persist after the removal of the tumor [6]. In our case, the follow-up after surgery does not show any persisting involvement of the heart.

Even though pheochromocytoma is a rare condition, if a young patient presents with unexplained cardiac dysfunction, we should consider pheochromocytoma [7]. The prognosis of pheochromocytoma-induced cardiac diseases depends on early diagnosis and treatment [8,9].

## Conclusions

Our case report provides valuable insights into considering pheochromocytoma as a potential cause of cardiac presentations with shock in young patients. Pheochromocytoma rarely presents with serious cardiac manifestations. Early diagnosis and timely intervention significantly improve patient mortality. The initial presentation of pheochromocytoma may vary, but with proper monitoring and follow-up, it becomes possible to diagnose it early.
